# Unrecognized Charcot-Marie-Tooth Disease Unmasked by Chemotherapy-Induced Neurotoxicity

**DOI:** 10.7759/cureus.113668

**Published:** 2026-07-30

**Authors:** Isabel V Fernandes, Sara Melo, Gabriela M Sousa, Maria Isabel Pazos, Cláudia Lima

**Affiliations:** 1 Medical Oncology, Instituto Português de Oncologia de Coimbra Francisco Gentil, Coimbra, PRT; 2 Neurology, Instituto Português de Oncologia de Coimbra Francisco Gentil, Coimbra, PRT

**Keywords:** breast cancer chemotherapy, charcot-marie-tooth disease, chemotherapy-induced peripheral neuropathy, neurotoxicity, pmp22 gene duplication

## Abstract

Chemotherapy-induced peripheral neuropathy (CIPN) is among the most prevalent and clinically significant toxicities from systemic cancer treatments, with taxanes being a leading cause. It is characterised by a length-dependent, predominantly sensory axonal neuropathy that can stabilise or improve after treatment discontinuation. However, the clinical course can be profoundly altered by underlying peripheral nerve pathology. We report the case of a 68-year-old woman with luminal B, HER2-negative breast cancer who developed severe peripheral neuropathy during adjuvant chemotherapy with doxorubicin/cyclophosphamide followed by weekly paclitaxel. Treatment was discontinued after 9 of 12 planned paclitaxel cycles due to grade 3 peripheral neuropathy ( Common Terminology Criteria for Adverse Events (CTCAE) v5.0). Despite cessation of the offending agent and initiation of duloxetine, symptoms continued to worsen, with progressive motor impairment and significant gait instability. Nerve conduction studies revealed a severe sensorimotor demyelinating polyneuropathy, atypical for the predominantly axonal pattern of taxane-induced CIPN. Inflammatory, infectious, paraneoplastic, and metabolic causes were excluded, and empirical corticosteroids and intravenous immunoglobulin proved ineffective. Genetic testing identified a peripheral myelin protein 22 (PMP22) duplication (17p11.2), confirming Charcot-Marie-Tooth disease type 1A (CMT1A), a hereditary demyelinating neuropathy previously subclinical. This case report underscores the value of serial clinical and electrophysiological assessment when neuropathy is atypical, disproportionately severe, or refractory to standard treatment.

## Introduction

Chemotherapy-induced peripheral neuropathy (CIPN) is a common and clinically significant toxicity of systemic cancer treatment, reported in up to 68% of patients receiving neurotoxic agents [1]. Taxanes are among the most frequently implicated agents, producing a predominantly sensory, length-dependent, axonal neuropathy through mechanisms involving microtubule dysfunction, mitochondrial injury, and disruption of axonal transport in dorsal root ganglion neurons [2]. CIPN manifests as distal, numb, and neuropathic pain, and severity is usually correlated with cumulative drug dose and treatment duration [2]. Current American Society of Clinical Oncology (ASCO) guidelines recommend duloxetine as the sole pharmacological agent with evidence supporting its use in painful CIPN, though its effect remains modest [3]. In cases of intolerable or functionally significant neuropathy, dose reduction, treatment delay, or discontinuation is advised [3].

Neuropathy developing during chemotherapy is usually attributed to CIPN. However, alternative diagnoses should be considered whenever neuropathy is atypical, motor predominant, disproportionately severe, or refractory to standard management; a distinction that is easily overlooked in routine oncological practice. A pre-existing hereditary neuropathy can substantially alter the severity, rate of progression, as well as electrophysiological signature of peripheral nerve injury, producing a clinical picture that departs markedly from what would be expected from chemotherapy alone.

Charcot-Marie-Tooth disease (CMT) is the most common hereditary neuropathy, with an estimated prevalence of 1 in 2500 individuals [4]. CMT1A, caused by duplication of the peripheral myelin protein 22 (PMP22) gene on chromosome 17p11.2, is the most frequent subtype, accounting for approximately 40-70% of all CMT cases and characterised by a slowly progressive, symmetric, sensorimotor demyelinating polyneuropathy [4,5]. CMT1A displays highly variable clinical expressivity. Many carriers remain asymptomatic or show only subtle findings, such as pes cavus, reduced deep tendon reflexes, or mild distal weakness, for decades without a formal diagnosis [4,5].

In this population, neurotoxic chemotherapy acts as a trigger, unmasking and severely exacerbating subclinical hereditary neuropathy [6,7]. Vincristine and, to a lesser extent, paclitaxel are the agents most consistently associated with atypical and disproportionately severe peripheral neurotoxicity in patients with undiagnosed CMT [6]. In most affected patients described in the literature, no prior diagnosis of CMT existed at the time of chemotherapy initiation, and targeted retrospective family history assessment subsequently disclosed signs of subclinical hereditary neuropathy in many cases [7].

## Case presentation

A 68-year-old woman, with no relevant medical history, was diagnosed with clinical stage II, per American Joint Committee on Cancer (AJCC) 8^th^ edition, luminal B-like, HER2-negative invasive ductal bifocal carcinoma of the breast on routine screening mammography. She underwent a modified radical mastectomy with sentinel node biopsy, staging as pT3N1M0, a pathological stage IIIA (AJCC 8^th^ edition [8]). Due to the large tumor size, positive axillary lymph nodes, and luminal B-like subtype, adjuvant chemotherapy was recommended. Treatment was initiated with 4 cycles of doxorubicin 60 mg/m² and cyclophosphamide 600 mg/m² (AC), followed by 12 planned cycles of weekly paclitaxel 80 mg/m².

After the first cycle of paclitaxel, the patient reported bilateral distal paraesthesia in the lower limbs, initially described as mild, symmetrical tingling in the feet. She had no prior neurological symptoms or history of alcohol consumption or diabetes. Her body mass index (BMI) was 24 Kg/m^2 ^and her Eastern Cooperative Oncology Group Performance Status (ECOG-PS [9]) was 0 prior to taxane therapy and 1 at this point. She also denied any prior sensory disturbance or motor impairment and had no known family history of neuropathy. By this time, duloxetine was initiated in accordance with ASCO guideline recommendations [3] but produced no meaningful benefit. Over subsequent cycles, symptoms worsened progressively and disproportionately, involving the hands and increasing in intensity. Paclitaxel was discontinued after 9 of 12 planned cycles due to grade 3 peripheral neuropathy per CTCAE v5.0 [10], characterised by severe symptoms limiting self-care activities of daily living.

**Figure 1:**
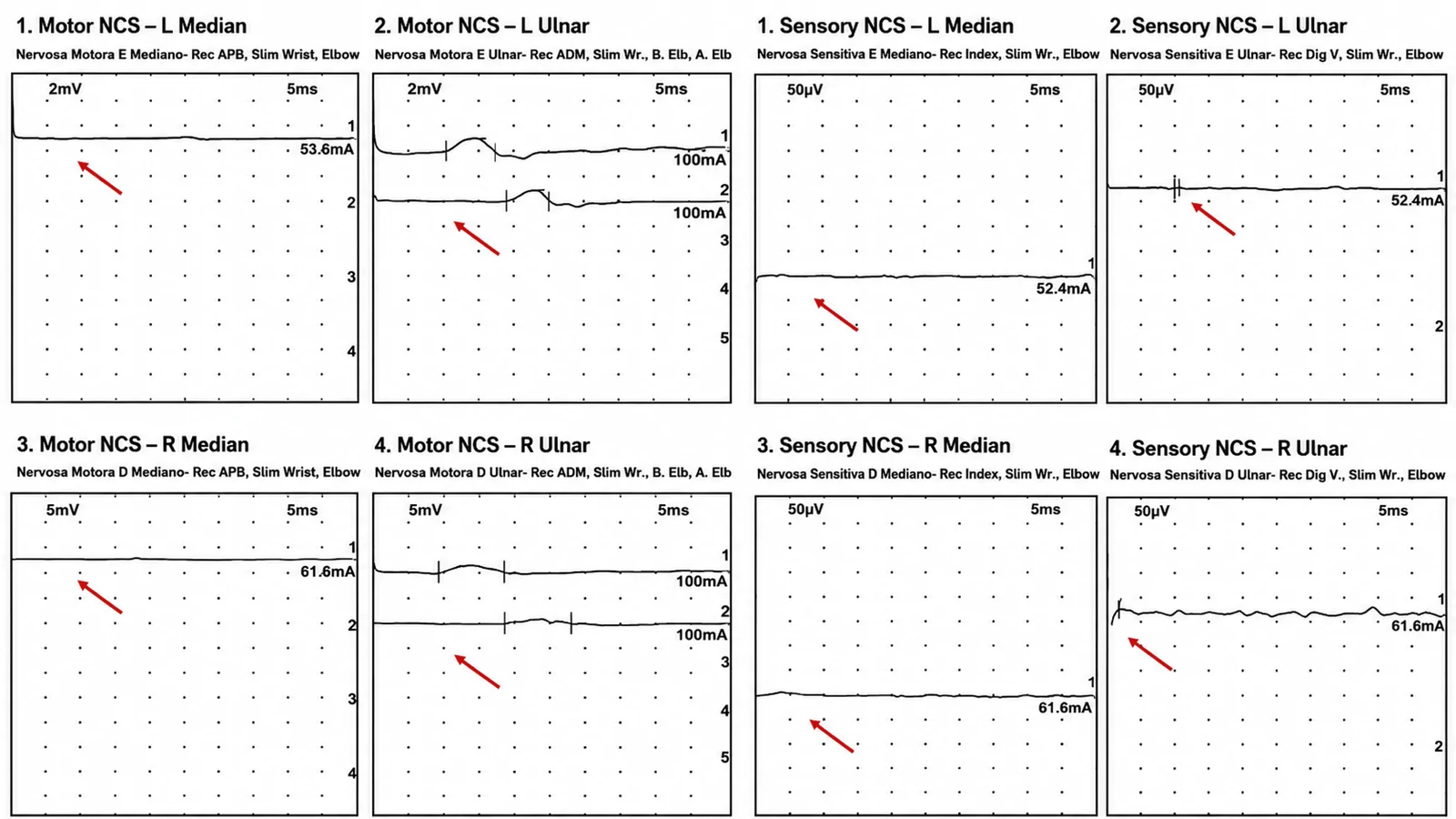
Representative motor and sensory nerve conduction waveform tracings, corresponding to the nerves and sites detailed in Table 1 Arrows indicate the main abnormal finding in each tracing, including the unobtainable (NR) median nerve responses. L, left; NCS, nerve conduction studies; R, right

Despite treatment cessation, the patient's neurological status continued to deteriorate over the following months, with increasingly painful acroparaesthesia in all four limbs, progressive weakness of the intrinsic muscles of the feet and hands, and significant gait instability necessitating the use of a walking aid. Neurological examination revealed absent deep tendon reflexes at the ankles and knees bilaterally, globally reduced tendon reflexes in the upper limbs, and markedly impaired vibration sense at the medial malleoli and dorsum of the hands. Motor examination demonstrated distal weakness, more pronounced in the lower limbs, with bilateral foot drop. Nerve conduction studies and electromyography demonstrated a severe sensorimotor demyelinating polyneuropathy, with markedly and uniformly reduced motor and sensory conduction velocities across multiple nerve segments, substantially prolonged distal latencies, and evidence of conduction block in several territories (Table 1 and Figure 1). This demyelinating profile was discordant with the predominantly axonal involvement expected in taxane-induced CIPN [2].

**Table 1:** Motor and sensory nerve conduction studies showing markedly reduced conduction velocities and prolonged distal latencies in both ulnar nerves, with bilaterally unobtainable median nerve responses AMP, amplitude; CV, conduction velocity; Dif., difference (interlatency interval); Dist., distance; Dur., duration; L, left; Lat1, onset latency; Lat2, peak latency; N, normal; NR, no response; R, right; SC, supraclavicular; Rec, recording site; Stim, stimulation site; B.Elb, below elbow; A.Elb, above elbow Note: Values in bold are significant.

Motor nerve conduction							
Nerve / Site	Lat1 (ms)	Dur. (ms)	AMP (mV)	Area (mVms)	Dist. (mm)	Dif. (ms)	CV (m/s)
L Ulnar - Rec ADM, Stim Wrist, B.Elb, A.Elb, Ax, SC							
Wrist	10.9 (N≤3.3)	6.7	1 (N≥6.0)	2.2	70	10.9	-
Distal elbow	18.9 (N≤3.3)	6.3	0.8 (N≥6.0)	3.1	170	8	21 (N≥49)
R Ulnar - Rec ADM, Stim Wrist, B.Elb, A.Elb, Ax, SC							
Wrist	9.3 (N≤3.3)	9.2	1.5 (N≥6.0)	4.7	70	9.3	-
Distal elbow	18.5 (N≤3.3)	9.5	0.9 (N≥6.0)	4.2	195	9.2	21 (N≥49)
L Median - Rec APB, Stim Wrist, Elbow, Axilla, SC							
Wrist	NR (N≤4.4)	NR	NR (N≥4.0)	NR	70	NR	-
R Median - Rec APB, Stim Wrist, Elbow, Axilla, SC							
Wrist	NR (N≤4.4)	NR	NR (N≥4.0)	NR	70	NR	-
Sensory nerve conduction							
Nerve / Site	Lat1 (ms)	Lat2 (ms)	AMP (µV)	Dist. (mm)	Dif. (ms)	CV (m/s)	-
L Ulnar - Rec Dig V, Stim Wrist, Elbow (Antidromic)							
Wrist	10	10.6 (N≤3.1)	5 (N≥10)	140	10	14 (N≥50)	-
R Ulnar - Rec Dig V, Stim Wrist, Elbow (Antidromic)							
Wrist	1.7	2 (N≤3.1)	5 (N≥10)	140	1.7	81 (N≥50)	-
L Median - Rec Index, Stim Wrist, Elbow (Antidromic)							
Wrist	NR	NR (N≤3.5)	NR (N≥20)	140	NR	NR (N≥50)	-
R Median - Rec Index, Stim Wrist, Elbow (Antidromic)							
Wrist	NR	NR (N≤3.5)	NR (N≥20)	140	NR	NR (N≥50)	-

Given this critical electrophysiological discrepancy and the progressive clinical course despite treatment withdrawal, an extensive and systematic diagnostic workup was undertaken.

Lumbar puncture yielded acellular cerebrospinal fluid (CSF) with mildly elevated total protein, without pleocytosis, precipitating a trial of immunomodulatory therapy - intravenous methylprednisolone 1 g per day for 5 days followed by two cycles of intravenous immunoglobulin at 2 g/kg - given the concern for chronic inflammatory demyelinating neuropathy. No clinical benefit was observed, a finding that argued against an inflammatory or immune-mediated aetiology. CSF and serum onconeural antibody panels, including anti-Hu, anti-Yo, anti-Ri, anti-amphiphysin, anti-CV2, and anti-NMDAR, were negative. A comprehensive laboratory workup, including thyroid function, vitamin B12 and B6, folic acid, serum protein electrophoresis, antinuclear antibodies, antineutrophil cytoplasmic antibodies, cryoglobulins, and a systemic vasculitis panel, was unremarkable. Infectious disease screening for HIV, *Borrelia burgdorferi*, and syphilis (VDRL/TPHA) was negative. Computed tomography (CT) of the chest, abdomen, and pelvis and whole-body positron emission tomography with fluorodeoxyglucose (¹⁸F-FDG PET) imaging confirmed stable oncological disease with no evidence of progression. A paraneoplastic aetiology was thus considered unlikely.

The persistence of neurological deterioration after chemotherapy withdrawal, combined with the demyelinating electrophysiological pattern, raised the suspicion for underlying hereditary neuropathy. Genetic testing was performed, revealing duplication of the PMP22 gene on chromosome 17p11.2, confirming a diagnosis of CMT1A. Prompted by this finding, a reassessment of the family history identified that a first-degree relative (the patient's daughter) had a longstanding history of mild bilateral pes cavus and intermittent stumbling, previously uninvestigated, and consistent with familial CMT with variable expressivity.

At the most recent re-evaluation, the patient remained clinically stable, with no further progression of the neurological deficits. Regarding the oncological disease, she was recommended to start adjuvant hormone therapy, which she is currently undergoing, while maintaining regular follow-up with both Medical Oncology and Neurology.

## Discussion

In this case, a pre-existing CMT1A, subclinical throughout the patient's adult life, was unmasked by paclitaxel. The presentation mimicked severe CIPN in its timing and distribution, but differed fundamentally in its progression after treatment cessation, a discrepancy that nerve conduction studies helped clarify. Beyond illustrating the diagnostic value of electrophysiological testing in atypical CIPN, this case highlights a clinically significant issue: chemotherapy can trigger clinically meaningful exacerbation of an unrecognised hereditary neuropathy, with direct consequences for treatment decisions, oncological follow-up, and family screening once the diagnosis is made.

The distinction between axonal and demyelinating neuropathy on nerve conduction studies represents a fundamental diagnostic bifurcation. While chemotherapy agents may very rarely cause demyelinating neuropathy (more frequent with bortezomib toxicity) [11], CIPN characteristically presents as a length-dependent axonal sensory neuropathy, with nerve conduction studies showing reduced or absent sensory nerve action potential amplitudes, while conduction velocities are relatively preserved [2]. In the present case, the electrophysiological findings were instead characterised by diffuse, uniform slowing of conduction velocities and markedly prolonged distal latencies - the electrophysiological signature of a primary demyelinating polyneuropathy, such as CMT1A [4,5]. This discordance was the diagnostic clue that prompted a search for an alternative aetiology. In our experience, the temptation to attribute all neuropathy during chemotherapy to CIPN is real, and the present case illustrates how this assumption, without electrophysiological corroboration, can delay the correct diagnosis.

The phenomenon of neurotoxic chemotherapy unmasking or exacerbating subclinical CMT is well-established in the literature, though it remains underappreciated in day-to-day oncological practice. The earliest and most influential evidence came from Graf et al., who described three families with CMT1A in which a family member receiving vincristine developed rapid-onset, severe, and largely irreversible neuropathy, establishing that the 17p11.2 duplication constitutes a major risk factor for vincristine neurotoxicity [12]. Subsequent case reports and series extended this risk to taxane-based regimens, including paclitaxel [6,13] and docetaxel [14]. A retrospective analysis from the OncoNeuroTox database identified eight patients with concomitant CMT among 428 with CIPN. In seven of eight, CMT had been unknown prior to chemotherapy, yet targeted retrospective family enquiry disclosed signs of subclinical hereditary neuropathy in five [7]. The pathophysiological basis for this vulnerability lies in the reduced functional reserve of the peripheral nervous system in CMT patients, where pre-existing dysmyelination and impaired axonal transport capacity render the nerve disproportionately susceptible to additional toxic insults [6,7]. This mechanism appears to have been operative in our patient, given the rapidity and severity of the neurological deterioration relative to the cumulative paclitaxel dose received.

The absence of benefit from high-dose corticosteroids and intravenous immunoglobulin in this case, while clinically frustrating in the short term, was diagnostically informative. CMT1A is a genetically determined structural abnormality of peripheral myelin, not an immune-mediated process, and is therefore inherently unresponsive to immunomodulatory treatments. The clinical course reinforced the need for genetic investigation. Therefore, in oncology-related neuropathy in which the initial working diagnosis of CIPN or inflammatory neuropathy is not supported by treatment response, genetic testing should be pursued promptly.

The diagnosis of CMT1A carries significant implications beyond the immediate clinical episode. CMT1A is inherited in an autosomal dominant pattern, with each first-degree relative carrying a 50% probability of harbouring the duplication [4,5]. Genetic counselling should be offered to the patient and their family. In terms of future oncological management, the heightened susceptibility of CMT patients to neurotoxic agents must be factored into chemotherapy selection. Where disease control requires systemic treatment, regimens with lower neurotoxic potential should be preferred when equivalent options exist. Published experience suggests that substituting paclitaxel with docetaxel or opting for non-taxane and non-vinca alkaloid regimens may reduce the risk of severe neurotoxicity in this population [13,14]. In any case, neurotoxic exposure in a CMT patient warrants close proactive neurological monitoring with low thresholds for dose modification.

This case raises the question of whether structured pre-chemotherapy neurological screening should be incorporated into routine clinical practice, particularly before initiating highly neurotoxic regimens. Although universal genetic screening is not currently recommended, a case can be made for targeted assessment in patients presenting with any subtle features suggestive of a pre-existing neuropathy such as reduced ankle reflexes, pes cavus, or a family history of unexplained gait difficulty or foot deformity. Had a structured neurological enquiry been performed before initiating paclitaxel in our patient, the possibility of a subclinical hereditary neuropathy might have been raised at the outset.

## Conclusions

Severe peripheral neuropathy arising during chemotherapy is not invariably attributable to CIPN. When the clinical course is disproportionately severe, progresses despite treatment cessation, or is unresponsive to appropriate symptomatic management, the diagnosis warrants reassessment. Electrophysiological characterisation is essential: a demyelinating pattern should prompt systematic exclusion of inflammatory, infectious, paraneoplastic, and hereditary causes. CMT1A, the most common hereditary neuropathy, may remain entirely subclinical into adulthood and be dramatically unmasked by neurotoxic chemotherapy. Genetic testing should be integrated earlier into the diagnostic algorithm in patients with atypical neuropathy presentations, particularly when there is a poor response to supportive therapy, and in those with subtle baseline neurological findings or a family history suggestive of peripheral nerve disease. Early diagnosis has practical consequences, as it spares patients from ineffective treatments and potential long-term impairment, guides future chemotherapy decisions, and opens the door to genetic counselling for first-degree relatives who may themselves be at risk.
